# An incomplete step in the right direction: Peru’s National Institute of Health establishes cost-effectiveness threshold

**DOI:** 10.11606/s1518-8787.2022056004923

**Published:** 2022-11-18

**Authors:** Enrique M. Saldarriaga

**Affiliations:** I University of Washington CHOICE Seattle WA United States University of Washington. The Comparative Health Outcomes, Policy, and Economics (CHOICE) Institute. Seattle, WA, United States

Seattle, June 20, 2022

Revista de Saúde Pública

Dear Editor,

The Peruvian National Institute of Health (INS – *Instituto Nacional de Salud del Perú*) established for the first time in the country a cost-effectiveness (CE) threshold for the analysis of interventions, health technologies and pharmaceutical products^1^. This threshold is an essential element for investment decision-making as it signals which interventions are most likely to create benefits above their implementation and maintenance costs. The INS determined that an intervention will be cost-effective if its cost per unit of health gained is between 2.2 and 4.4 times the value of a tax unit (UIT – *Unidad Impositiva Tributaria*) valued at 4,600PEN in 2022^2^; in dollars, between 2,663 and 5,326USD. In the following paragraphs I present why I believe this is a step in the right direction albeit incomplete.

This decision is appropriate for three reasons. First, it provides an objective, quantifiable, and standard element of analysis for health investments decision-making. In addition, it facilitates the comparison of economic feasibility between interventions that would not be comparable otherwise. Second, by establishing the range based on an independent measure (the UIT) that is updated annually based on macroeconomic criteria, it increases the probability of being sustained over time and of being adopted by the scientific community. Third, the range established is in line with the growing literature that strongly criticizes the use of GDP-based thresholds as being insufficiently responsive to the opportunity cost of public investment in settings (i.e., countries, regions) with limited access to resources and therefore with a higher need for prioritization of resources^3,4^. In a recent study, Kazibwe et al.^5^ analyzed 197 health economic evaluations and found that all those from Latin America used CE thresholds based on the country’s GDP per capita. The stipulation of the INS is thus a pioneering step in Latin America, where the literature on methodologies for health technology assessment is scarce and shows a preference for thresholds based on GDP^6^.

Despite these attributes, the range has some limitations. First, although there is debate about the best ways to use and calculate the CE threshold, in practice there is agreement that it should represent the opportunity cost of financing an intervention from the healthcare sector or societal perspectives^7^. Thus, it is not clear what is the logic behind linking the CE threshold to the UIT. It is not evident what is the relationship of this tax unit with Peru’s health sector budget, the technical or financial capacity of the health provider, or the preferences of the population; which would have been expected of an instrument that seeks to serve as an investment guide. The UIT is an instrument used in taxation and therefore correlated with economic activity in general, but not necessarily with the spending capacity of the health sector. Second, the INS’s statement does not describe which health unit was considered during the establishment of the threshold. This is relevant because the health consequences in economic evaluations can be expressed in different health units – usually years of life gained, quality of life-adjusted health years (QALYs) or years lost due to disability (DALYs) – that represent different types of public health gains and therefore willingness to invest. While the INS directive would not be expected to establish which health unit researchers and health technology assessment officers should use, because that is their prerogative, it could provide further instructions for an appropriate usage. The immediate consequence of the lack of clarity in this regard is that interventions using only life-years to demonstrate effectiveness will look much more cost-effective than interventions using comprehensive measures of health production such as QALYs and DALYs.

In conclusion, the establishment of a range of CE by the INS is a very important tool for the system of technological evaluations in Peru. However, its adoption in the long-term by the scientific community will depend on being able to explain the correlation between the UIT and the willingness to pay or spending capacity of the health system for improvements in health outcomes, as well as having more information on what health outcome was considered for its determination and usage.

## References

[1] Ministerio de Salud (PE) (2022). Resolución Ministerial N° 159-2022/MINSA, de 4 de marzo de 2022. Establecer el umbral de costo - efectividad de las evaluaciones de tecnologías sanitarias realizadas por la Dirección General de Medicamentos, Insumos y Drogas y por el Instituto Nacional de Salud.

[2] Ministerio de Economia y Finanzas (PE) (2021). Decreto Supremo Nº 398-2021EF, de 30 de diciembre de 2021. Valor de la Unidad Impositiva Tributaria durante el año 2022.

[3] Woods B, Revill P, Sculpher M, Claxton K (2016). Country-level cost-effectiveness thresholds: initial estimates and the need for further research. Value Health.

[4] Revill P, Walker S, Madan J, Ciaranello A, Mwase T, Gibb DM, Claxton K (2014). Using cost-effectiveness thresholds to determine value for money in low-and middle-income country healthcare systems: are current international norms fit for purpose?.

[5] Kazibwe J, Gheorge A, Wilson D, Ruiz F, Chalkidou K, Chi YL (2022). The use of cost-effectiveness thresholds for evaluating health interventions in low- and middle-income countries from 2015 to 2020: a review. Value Health.

[6] Augustovski F, Garay OU, Pichon-Riviere A, Rubinstein A, Caporale JE (2010). Economic evaluation guidelines in Latin America: a current snapshot. Expert Rev Pharmacoecon Outcomes Res.

[7] Thokala P, Ochalek J, Leech AA, Tong T (2018). Cost-effectiveness thresholds: the past, the present and the future. Pharmacoeconomics.

