# Supplementary material for: An incomplete step in the right direction: Peru’s National Institute of Health establishes cost-effectiveness threshold
Source: Rev Saude Publica. 2022 Nov 18;56:106. doi: 10.11606/s1518-8787.2022056004923 (PMC9749660; doi:10.11606/s1518-8787.2022056004923)
Supplement: Translation

# Translation: Un paso incompleto en la dirección correcta: el Instituto Nacional de Salud del Perú establece el rango de costo-efectividad

**Authors:** Enrique M. Saldarriaga

**Affiliations:** I United States University of Washington. The Comparative Health Outcomes, Policy, and Economics (CHOICE) Institute. Seattle, WA, United States

Seattle, 20 de junio de 2022

Revista de Saúde Pública

Estimado Editor,

El Instituto Nacional de Salud del Perú (INS) estableció por primera vez en el país un umbral de costo-efectividad (CE) para el análisis de intervenciones y tecnologías de la salud y productos farmacéuticos^1^. Dicho umbral es un elemento esencial para la toma de decisiones de inversión pues indica qué intervenciones tienen mayor posibilidad de crear beneficios por encima del costo de su implementación y mantenimiento. El INS estableció que una intervención será costo-efectiva si su costo por unidad de salud ganada es entre 2.2 y 4.4 veces el valor de una unidad impositiva tributaria (UIT) valorizada en 4,600PEN en 2022^2^ - equivalente a entre 2,663 y 5,326USD. A continuación, expongo por qué considero que este es un paso en la dirección correcta pero incompleto.

Esta decisión es acertada por tres razones. Primero, es un elemento de análisis objetivo, cuantificable y estándar para la toma de decisiones en inversiones de salud. Adicionalmente, facilita la comparación de factibilidad económica entre intervenciones que de otra forma no serían comparables. Segundo, al establecer el rango en función de una medida (la UIT) independiente y que se actualiza anualmente en función a criterios macroeconómicos, incrementa la probabilidad de ser sostenida en el tiempo y de ser adoptada por la comunidad científica. Tercero, el rango establecido está en concordancia con la creciente literatura que critica fuertemente el uso de rangos basados en el PBI por ser poco sensibles al costo de oportunidad de inversión pública en lugares (países, regiones) con acceso limitado a recursos y que por tanto tienen una necesidad elevada de priorización de recursos^3,4^. En un estudio reciente, Kazibwe et al.^5^ analizaron 197 evaluaciones económicas en salud y encontraron que todas aquellas provenientes de Latino América utilizaron umbrales de CE basados en el producto bruto interno (PBI) per cápita del país. La estipulación del INS es entonces también un paso pionero en Latino América, donde la literatura sobre metodologías para la evaluación de tecnologías sanitarias es escasa y muestra una preferencia por umbrales basados en PBI^6^.

Sin mengua de estos atributos, el establecimiento de este rango tiene algunas deficiencias. Primero, aun cuando existe debate respecto a las mejores formas de uso y cálculo del umbral de CE, en la práctica hay conceso de que debe representar el costo de oportunidad para el proveedor de salud o la sociedad de financiar una intervención^7^. Siendo así, no es claro cuál es la lógica detrás de atar el umbral de CE a la UIT, puesto que no es evidente la relación con el presupuesto de salud del Perú, la capacidad técnica o financiera del proveedor de salud, o las preferencias de la población, como se esperaría de un instrumento que busca fungir de guía de inversiones. La UIT es un instrumento usado en tributación y por lo tanto correlacionado con la actividad económica en general, pero no necesariamente con la capacidad de gasto del sector salud. En segundo lugar, la Norma no establece parámetros para el uso del rango de CE. En particular, qué unidad de salud se consideró durante el establecimiento del umbral. Esto es relevante porque las evaluaciones económicas se pueden expresar en diversas unidades de salud – usualmente años de vida ganados, años de salud ajustados por calidad de vida (AVAC) o perdidos por discapacidad (AVAD) – que representan distintos tipos de ganancia en salud pública y por consiguiente disposición a invertir. Si bien no se esperaría que la Norma del Ministerio de Salud del Perú (MINSA) establezca qué unidad de salud utilizar, pues eso es discreción de los investigadores, sí podría establecer parámetros para su uso. La consecuencia inmediata de la falta de claridad en este sentido es que intervenciones que utilicen únicamente años de vida para demostrar efectividad van a parecer mucho más costo-efectivas que intervenciones que utilizan medidas comprehensivas de producción de salud como AVAC y AVAD.

En conclusión, el establecimiento de un rango de CE por parte del MINSA es una herramienta muy importante para el sistema de evaluaciones tecnológicas en el Perú. No obstante, su adopción en la comunidad científica y su uso en el largo plazo dependerá de ser posible explicar la correlación entre la UIT y la disposición a pagar o capacidad de gasto del sistema de salud por mejoras en los resultados de salud, así como de tener mayor información sobre qué resultado de salud se tuvo en cuenta para la determinación del umbral.
